# Seroprevalence of Pestivirus Infection and Associated Risk Factors in Sheep in Konya Province, Türkiye

**DOI:** 10.3390/vetsci13080812

**Published:** 2026-08-16

**Authors:** Ömer Barış İnce, Ahmet Sait, Alparslan Ayaz

**Affiliations:** 1 Department of Virology, Faculty of Veterinary Medicine, Necmettin Erbakan University, 42310 Konya, Türkiye; 2 Viral Diagnostic Laboratory, Pendik Veterinary Control Institute, 34890 Pendik, Türkiye; saitvet@gmail.com; 3 Ministry of Agriculture and Forestry, Karaman Province Directorate, 70100 Karaman, Türkiye; dralpayaz@gmail.com

**Keywords:** sheep, pestivirus, border disease virus, RT-PCR, antibody-antigen ELISA, herd, animal health, animal welfare

## Abstract

Pestivirus infections, including those caused by Border disease virus (BDV), are associated with reproductive disorders, neonatal losses, and persistent infections in sheep, potentially resulting in substantial economic losses. This study aimed to determine the animal- and herd-level pestivirus seroprevalence among sheep in Konya Province, Türkiye, to investigate evidence of current infection, and to identify factors associated with seropositivity. Serum and whole-blood samples were collected from 280 sheep in 16 herds. Serum samples were tested for pestivirus antibodies by competitive ELISA, while whole-blood samples were examined for pestiviral antigen and RNA by antigen ELISA and real-time RT-PCR, respectively. Epidemiological associations were evaluated using generalized estimating equations (GEE), with herd included as the clustering variable. Pestivirus antibodies were detected in 168 of 280 sheep, corresponding to an animal-level pestivirus seroprevalence of 60.00% (95% confidence interval (CI): 54.16–65.57). At the herd level, 14 of 16 herds (87.5%) had at least one seropositive animal, while herd-specific antibody seropositivity ranged from 0% to 82.76%. Four animals were antigen-reactive at the initial sampling, and one remained so at the 15-day retest; however, all samples were negative by real-time RT-PCR. Therefore, persistent infection could not be confirmed. In the multivariable GEE model, animal age, herd disease history, and the introduction of new animals into the herd were significantly associated with pestivirus seropositivity. Together with previous studies from Türkiye, our findings indicate that pestivirus exposure remains common in sheep populations and warrants continued regional surveillance. Serological screening should therefore be complemented by direct detection methods, including antigen ELISA and real-time RT-PCR, when investigating current or persistent pestivirus infection. Informing herd owners about pestivirus infections and appropriate biosecurity measures may also contribute to reducing their potential impact on animal health and production.

## Simple Summary

Some viral infections in sheep can cause reproductive losses, weak lambs at birth, and reduced productivity, resulting in economic losses for farmers. This study aimed to determine the extent of sheep exposure to pestiviruses in Konya Province and to identify factors associated with seropositivity. Blood samples were collected from 280 sheep in 16 herds. Herd-specific pestivirus antibody seropositivity ranged from 0% to 82.76%, while the overall animal-level pestivirus seroprevalence was 60.00% (168/280). Pestivirus antigen was detected in four animals at the initial sampling; one of these animals remained antigen-reactive at the 15-day retest. However, all samples were negative by real-time RT-PCR. Pestivirus seropositivity was associated with animal age, herd disease history, and the introduction of new animals into the herd. These findings support the importance of quarantine, testing of newly introduced animals, and appropriate herd-level biosecurity measures to reduce pestivirus exposure and its potential economic impact.

## 1. Introduction

Pestiviruses, members of the family Flaviviridae, are enveloped, positive-sense, single-stranded RNA viruses that can have adverse effects on reproductive performance, growth, and overall health in domestic and wild animals. Border disease, caused primarily by viruses belonging to the species Pestivirus ovis, is an economically important viral disease of sheep and is associated with abortion, stillbirth, infertility, the birth of weak lambs, and the characteristic “hairy shaker lamb” syndrome [1,2,3,4]. Pestivirus ovis primarily infects sheep and, less frequently, goats [5,6,7]. However, the host specificity of pestiviruses is not absolute; sheep can be infected with border disease virus, bovine viral diarrhea virus, and other genetically distinct ruminant pestiviruses. This characteristic, especially in production systems where cattle and small ruminants are housed on the same farm or share common pastures, facilitates interspecies transmission and complicates the interpretation of serological findings at the pathogen level [8,9,10,11,12].

The clinical and economic significance of border disease largely stems from the consequences of infections occurring in pregnant ewes. In adult and non-pregnant sheep, acute infection is mostly subclinical or mild, whereas transplacental infection during pregnancy can lead to embryonic death, abortion, stillbirth, low birth weight, growth retardation, congenital anomalies, neurological signs, and the birth of weak lambs with abnormal fleece appearance [7,9,13,14]. If the fetus becomes infected before the development of immune competence, virus-specific immunotolerance can develop, leading to the birth of lambs that are persistently infected (PI) for life. These animals are mostly antibody-negative but have continuous viremia and are the main source of infection within the herd, perpetuating the infection by releasing large amounts of the virus [7,15]. The recent report of a persistently infected sheep in a migratory herd, confirmed by molecular methods, virus isolation, and re-sampling, highlights the importance of apparently healthy animals in the spread of infection between herds and regions [16].

The seroprevalence of pestivirus in sheep varies significantly between countries, regions, and farms; it has been reported that this difference may be related to factors such as the breeding system, animal age, herd size, farm type, geographical location, common pasture use, and animal movements [9,12,17]. Higher seropositivity in older animals can be explained by their greater likelihood of encountering infection over their lifespan. In addition, the introduction of external animals to the herd, uncontrolled animal movements, nomadic breeding, and the lack of quarantine measures can facilitate the entry of transient or persistently infected animals into susceptible herds [15,17].

The antigenic and genetic diversity of pestiviruses complicates the diagnosis and epidemiological assessment of infections in small ruminants. Antibody enzyme-linked immunosorbent assays targeting the conserved p80 or NS2-3 proteins are widely used to determine prior exposure of animals to pestiviruses and to assess seroprevalence at the population level. However, due to the antigenic cross-reactivity among pestiviruses, antibody positivity alone cannot identify the virus species responsible for the infection. Serology indicates previous exposure, whereas antigen and nucleic acid detection methods allow investigation of active or persistent infection at the time of sampling. Therefore, it is recommended that serological screenings be supported by antigen detection, real-time reverse transcription polymerase chain reaction, retesting, and sequence analysis when necessary [7,15].

The presence of pestivirus infections in sheep in Turkey has been previously demonstrated through serological and molecular studies; genetically distinct sheep pestiviruses originating from Turkey have also been identified [18,19,20]. However, regional studies evaluating seroprevalence and associated risk factors at both the animal and herd levels are limited. In previous studies conducted in Konya province, one of Turkey’s important small ruminant farming centers, high seropositivity rates were reported in sheep herds [18], but risk factor studies where serological findings were evaluated alongside antigen and molecular methods have been limited. Moreover, variables including age, the herd’s disease history, and the incorporation of new animals into the herd have been identified as correlates of pestivirus seropositivity [21]. This study aimed to determine the seroprevalence of pestivirus in sheep in Konya Province, to investigate the presence of pestivirus antigen and viral RNA, and to evaluate animal-level factors associated with seropositivity. It is expected that the findings will contribute to planning regional surveillance and biosecurity practices based on scientific data.

## 2. Materials and Methods

### 2.1. Ethical Approval

The study protocol was approved by the Ethics Committee of the Experimental Animals Production and Research Center of the Faculty of Veterinary Medicine, Selçuk University (Report No: 2022/97, approval date: 25 August 2022). All animal procedures were performed in accordance with international guidelines for animal welfare and the ethical use of animals.

### 2.2. Study Area, Sampling Strategy, and Data Management

This study was conducted between 2022 and 2024 in six districts (Emirgazi, Ereğli, Karapınar, Seydişehir, Ilgın, and Selçuklu) of Konya Province, one of the major small-ruminant production regions in Türkiye. According to 2021 data from the Republic of Türkiye Ministry of Agriculture and Forestry, Konya Province had a small-ruminant population of 3,058,681 animals and 17,599 registered small-ruminant holdings [22]. The sampling design included both herd-level and animal-level components. The total number of herds to be included in the study was set at 16 based on field feasibility and logistical considerations. To ensure proportional representation across the predefined strata, the 16 were allocated to strata proportionally to the number of holdings in each stratum, using proportional stratified allocation and the formula nh = (Nh/N) × n, where nh represents the number of herds allocated to stratum h (district), Nh the number of eligible holdings in stratum h, N the total number of holdings in the sampling frame, and n the total number of herds to be sampled. Following proportional allocation, herds were randomly selected from the sampling frame within each stratum. The owners of the selected herds were subsequently informed about the study, and sampling was performed only after written informed consent had been obtained [23,24]. The minimum animal-level sample size was calculated using OpenEpi. Based on a population size of 3,058,681 small ruminants, an expected prevalence of 50%, an absolute precision of ±10%, a confidence level of 99.9%, and a design effect of 1.0, the minimum required sample size was calculated as 271 animals. To exceed this minimum requirement, a total of 280 sheep were included in the study [25,26]. Within each selected herd, predefined eligibility criteria were first applied, and individual sheep were then randomly selected from among the eligible animals. All sampled sheep originated from herds with no history of vaccination against pestiviruses. According to the predefined eligibility criteria, lambs younger than three months of age were excluded to minimize the potential influence of maternally derived antibodies on serological results. Animals showing clinical signs of acute illness at the time of sampling were also excluded. Blood samples were collected from the jugular vein of 280 sheep and transported to the laboratory under cold-chain conditions (+4 °C) using both coagulated and anticoagulated tubes. During field visits, face-to-face interviews with animal owners were conducted to record epidemiological data, such as herd size; animal mobility (introduction of external animals into the herd); herd composition (presence of cattle and goats); management style (pasture-based or residential); and past clinical disease history (abortions, respiratory or digestive system problems).

### 2.3. Laboratory Testing

#### 2.3.1. Antibody and Antigen ELISA

To detect specific antibodies against pestiviruses in serum samples, a commercial competitive ELISA kit targeting the non-structural p80 protein (ID Screen BVD p80 Antibody Competition assay, ID.vet-Innovative Diagnostics, Grabels, France) was used, and the results were evaluated according to the manufacturer’s instructions. The ID Screen BVD P80 Antigen Capture kit (ID.vet, Grabels, France) was used to detect pestivirus antigen. The test, based on the detection of viral antigens in study samples, was conducted according to the procedure described by the manufacturer. Animals that tested positive for the antigen were resampled and tested 15 days later to determine whether antigen positivity persisted.

#### 2.3.2. RNA Extraction and One-Step Real-Time RT-PCR

To validate the ELISA findings at the molecular level, viral RNA was isolated from leukocyte samples using the High Pure Viral Nucleic Acid Kit (Cat No.: 11 858 874 001, Roche, Mannheim, Germany). The extracted viral RNAs were analyzed by one-step real-time RT-PCR method with pan-pestivirus and species-specific (BVDV and BDV) primer-probe sets targeting the highly conserved 5′-UTR region of pestiviruses (Table 1). All real-time RT-PCR reactions were performed in multiplex format. The protocol described by La Rocca and Sandvik [27] was used for the detection of pestiviruses. Multiplex one-step real-time RT-PCR was performed using the one-step RT-PCR kit (RealTime Ready RNA Virus Master, Cat. No.: 05619416001, Roche) in a reaction mixture with a final volume of 20 µL, containing 4 µL RT-PCR buffer, 0.4 μL enzyme mix, 0.4 µM forward primer, 0.6 µM of equimolar amounts of two reverse primers, 0.5 µM of each probe, and 3 µL of sample RNA. Amplification was performed using a LightCycler 2.0 real-time PCR device (Roche Applied Science, Indianapolis, IN, USA) under the following conditions: a reverse transcription step at 50 °C for 8 min, an initial denaturation at 95 °C for 30 s, and 45 cycles, each consisting of 1 s at 95 °C and 30 s at 60 °C. Samples with a Ct value < 35 were considered positive. A known pestivirus-positive reference sample was included in every extraction batch and subsequent RT-PCR run to serve as both an extraction control and a positive amplification control.

### 2.4. Statistical Analysis

The statistical analyses of the epidemiological and laboratory data were performed using the GENLIN procedure in IBM SPSS Statistics version 26 and the geepack package (version 1.3.13) in R [28]. Potential factors associated with pestivirus seropositivity were first evaluated using univariable logistic GEE models, with herd specified as the clustering (repeated-subject) variable. Variables with *p* < 0.20 in the univariable GEE analyses were considered candidates and entered into the initial multivariable GEE model. Variables that were not statistically significant were subsequently removed, and variables with *p* < 0.05 were retained in the final model. Chi-square test or Fisher’s exact tests were used, as appropriate, compare categorical variables. To account for the correlation among animals sampled within the same herd, a multivariable GEE model was fitted, with herd specified as the clustering (repeated-subject) variable and the epidemiological variables obtained from the questionnaire included as independent variables. Candidate working correlation structures were compared using the Quasi-likelihood under the Independence Model Criterion (QIC), and an exchangeable working correlation structure was selected for the final model. The QIC of the final model was 474.086. Associations were expressed as regression coefficients (B), standard errors (SE), odds ratios (OR), 95% confidence intervals (CI), and *p*-values. Statistical significance in the final model was defined as *p* < 0.05.

## 3. Results

### 3.1. Laboratory Findings

Of the 280 sera tested by antibody ELISA, 168 were seropositive. The animal-level pestivirus seroprevalence was 60.00% (168/280; 95% CI: 54.16–65.57). At least one seropositive animal was detected in 14 of the 16 herds, corresponding to a herd-level prevalence of 87.5%. Herd-specific antibody seropositivity ranged from 0.00% to 82.76%. The association between age group (1–3 years and >3 years) and seropositivity was statistically significant (χ^2^ = 22.84, df = 1; *p* < 0.001) (Table 2).

Among the 280 whole-blood samples initially examined by antigen ELISA, four samples (1.43%) were antigen-reactive. All four antigen-reactive animals were negative for pestivirus antibodies. These animals were resampled 15 days later, and one of the four remained antigen-reactive; however, all samples were negative by real-time RT-PCR for pestivirus RNA. Therefore, persistent infection could not be confirmed in this repeatedly antigen-reactive animal. The pestivirus-positive reference control showed the expected amplification in all RT-PCR runs. Detailed herd-level results are presented in Table 3.

### 3.2. GEE Analysis Findings

The findings of the univariable GEE analyses are presented in Table 4. Variables meeting the predefined screening criterion (*p* < 0.20) were retained as candidate predictors for inclusion in the multivariable GEE model. After multivariable adjustment, age group, herd disease history, and the introduction of new animals remained statistically significant and were retained in the final model (Table 5). Compared with sheep aged >3 years, sheep aged 1–3 years had lower odds of seropositivity (OR = 0.61; 95% CI: 0.37–0.99; *p* = 0.04). Herds with no reported history of clinical disease had lower odds of pestivirus seropositivity than herds with a disease history (OR = 0.55; 95% CI: 0.33–0.91; *p* = 0.02). In contrast, the introduction of new animals into the herd was associated with 2.20-fold higher odds of pestivirus seropositivity compared with herds in which no new animals had been introduced (OR = 2.20; 95% CI: 1.22–3.97; *p* < 0.05).

## 4. Discussion

Pestivirus infections cause significant economic losses globally in small ruminant farming due to reproductive disorders, lamb losses, and cellular immune suppression in infected herds [29]. To develop effective control and eradication strategies, it is critically necessary to analyze the field prevalence of the infection and the risk factors that drive its spread dynamics [30]. In the present study, pestivirus antibodies were detected in 60.00% (168/280) of the sampled sheep, while herd-specific seropositivity ranged from 0% to 82.76%. Additionally, at least one seropositive animal was detected in 14 of the 16 herds, corresponding to a herd-level prevalence of 87.5%, indicating that pestivirus exposure was widespread but heterogeneously distributed among the sampled herds. Reported pestivirus seroprevalence in sheep varies widely depending on the geographical region, animal population, age distribution, herd management, sampling design, and serological test used. In the literature, it has been reported that the seroprevalence in sheep can range from 5% [31] to as high as 30% to 98% depending on the geographical region and management conditions [9]. In a comprehensive study conducted in Kazakhstan, the seroprevalence in small ruminants was found to be 53.7% [17], which is consistent with the data reported in other recent studies in the Mediterranean basin [15,32,33]. Differences between studies indicate that results should not be compared solely by country or region; rather, comparisons should take into account the age of sampled animals, herd selection, herd infection history, and breeding practices.

Persistently infected animals play a central role in pestivirus epidemiology because they may continuously shed large amounts of virus, and even a single PI animal may be sufficient to sustain within-herd transmission and contribute to interspecies transmission on shared pastures [16,34]. In the present study, four animals (1.43%) were antigen-reactive at the initial sampling, and one remained antigen-reactive at the 15-day retest. However, all samples were negative by real-time RT-PCR. Because the retesting interval was shorter than the minimum three-week interval recommended by WOAH, and because molecular confirmation was not obtained, persistent infection could not be confirmed in this repeatedly antigen-reactive animal. Therefore, PI prevalence was not estimated in the present study.

All samples, including the four initially antigen-reactive animals, were negative by real-time RT-PCR targeting the 5′-UTR. The reason for this discordance could not be determined definitively. Possible explanations include low viral RNA concentrations near or below the analytical detection limit, RNA degradation during sample collection, transport, or processing, co-extracted PCR inhibitors, or non-specific/or false-positive reactivity in the antigen ELISA. Primer/probe mismatch associated with pestivirus genetic diversity may also affect molecular detection [35]. The pestivirus-positive reference control showed the expected amplification in all RT-PCR runs, providing evidence against systematic extraction or amplification failure; however, sample-specific limitations cannot be completely excluded. Therefore, the available data do not allow a definitive explanation for the antigen-reactive/RT-PCR-negative findings.

The multivariable GEE model identified several factors associated with pestivirus seropositivity. A statistically significant association (*p* = 0.04) was observed between animal age and pestivirus seropositivity. The odds of seropositivity among animals aged 1–3 years were 0.61 times those of animals aged >3 years, suggesting greater cumulative exposure to pestiviruses with increasing age. This finding is consistent with previous reports indicating higher seropositivity in older animals [32,36]. Regarding herd management and biosecurity, the introduction of animals from outside the herd was associated with a 2.20-fold increase in the odds of pestivirus seropositivity (OR: 2.20; *p* < 0.05). Replacement animals (especially rams or breeding females) joining the herd without undergoing quarantine and virological screening can introduce infection into the herd. Uncontrolled animal movements may therefore represent an important biosecurity vulnerability for pestivirus transmission [21,34,37,38,39,40].

Furthermore, herds with no reported history of other clinical diseases had lower odds of pestivirus seropositivity than herds with a disease history (OR = 0.55; *p* = 0.02). This finding indicates an association between reported herd disease history and pestivirus seropositivity. Pestiviruses are known to modulate the host immune response through viral proteins, including Npro and Erns, and alterations in innate and cellular immune functions have been described [14,41,42,43]. However, because of the cross-sectional design of the present study, the direction and biological mechanism underlying the observed association between herd disease history and pestivirus seropositivity cannot be determined. When the present findings and the available literature are considered together, pestivirus exposure in sheep herds may be associated with animal age, animal movements, and herd management practices. Therefore, screening of newly introduced animals into the flock by Ag-ELISA or RT-PCR, identification and appropriate management of animals confirmed as persistently infected by repeat direct testing, and implementation of herd-based biosecurity procedures may contribute to reducing the adverse effects of pestivirus infections.

However, the study has some limitations. First, the relatively small number of herds included in the study (*n* = 16) limits the epidemiological representativeness of the sample at regional and national levels. Although GEE was used to account for within-herd correlation, robust standard error estimates may be less stable when the number of clusters is small, particularly for herd-level predictors. Therefore, the observed associations should be interpreted as exploratory and require confirmation in studies that include a larger number of herds. Second, because the study used a cross-sectional design, the relationships between the investigated factors and seropositivity identified in the multivariable analyses do not establish causality. Third, because the antibody ELISA used for serological screening cannot distinguish among pestivirus species owing to antigenic cross-reactivity, it was not possible to determine whether the observed seropositivity reflected exposure to Pestivirus ovis, BVDV, or another ruminant pestivirus, as molecular characterization could not be performed. Fourth, important management variables included in the model, such as herd disease history and the introduction of new animals, were based on herd owners’ reports and may, therefore, be subject to recall bias. In addition, the 15-day interval used for antigen retesting was shorter than the minimum three-week interval recommended for confirmation of persistent infection; therefore, PI status could not be established. Future studies should include larger and more diverse herd populations, longer repeat-testing intervals, and molecular characterization to validate these exploratory findings and improve their generalizability.

## 5. Conclusions

This study demonstrated widespread pestivirus exposure among sheep in Konya Province, with an animal-level seroprevalence of 60.00% and a herd-level prevalence of 87.5%. Pestivirus seropositivity was significantly associated with animal age, herd disease history, and the introduction of new animals into the herd. These associations should be interpreted as exploratory findings, particularly given the relatively small number of herds included in the study. Four animals were antigen-reactive at the initial sampling, and one remained antigen-reactive at the 15-day retest. However, all samples were negative by real-time RT-PCR, and persistent infection could not be confirmed. Therefore, serological evidence of pestivirus exposure should not be interpreted as evidence of current infection or infection with a specific pestivirus species. Serological findings should be evaluated together with direct detection methods, including antigen ELISA and real-time RT-PCR. Future studies that include larger numbers of herds, longer repeat-testing intervals, comprehensive molecular testing, virus isolation, and sequence analysis may help characterize circulating pestiviruses and support the development of risk-based surveillance and control strategies.

## Figures and Tables

**Table 1:** Primers and probes.

Primer-Probe	Sequence (5-3) ^a^	Genome Position	Specificity
106-F	CCATRCCCDTAGTAGGACTAGC	106–127 ^b^	BVDV/BDV
190-R	GYGTCGAACCAYTGACGACT	190–209 ^b^	BVDV
179-R	GYGTYGAACTACTGACGACT	179–198 ^c^	BDV
P-162	FAM-TGGATGGCYKAABCCCTGAGTACAG-EDQ	162–186 ^b^	BVDV
P-128	YY-ACTAGCYDTCGTGGTGAGATCCCTG-EDQ	128–152 ^c^	BDV

FAM: 6-carboxyfluorescein; YY: Yakima Yellow; EDQ: Eclipse Dark Quencher. IUPAC ambiguity codes: R = A/G; Y = C/T; D = A/G/T; K = G/T; B = C/G/T. ^a^ Reverse primers 190-R and 179-R complementary to positive sense target strand. ^b^ Pestivirus bovis NADL (GenBank accession M31182). ^c^ Pestivirus ovis X818 (GenBank accession FJ040215). All reactions were performed in multiplex format.

**Table 2:** Association between age group and pestivirus seropositivity in sheep.

Age Group	Number of Samples	Positive	Negative	Percentage (%)
1–3 years	96	39	57	40.63
>3 years	184	129	55	70.11
Total	280	168	112	60.00

*p* < 0.001. *X*^2^ = 22.84; df = 1.

**Table 3:** Herd-level distribution of pestivirus antibody and antigen ELISA results.

Herd	Sample Number	Ab-ELISA		95% CI	Ag-ELISA	
		Positive	Rate (%)		Reactive	Rate (%)
1	23	18	78.26	56.30–92.54	0	0.00
2	29	24	82.76	64.23–94.15	3	10.34
3	11	1	9.09	0.23–41.28	0	0.00
4	19	11	57.89	33.50–79.75	0	0.00
5	11	7	63.64	30.79–89.07	0	0.00
6	16	10	62.50	35.43–84.80	0	0.00
7	14	7	50.00	23.04–76.96	0	0.00
8	21	12	57.14	34.02–78.18	0	0.00
9	22	13	59.09	36.35–79.29	0	0.00
10	21	16	76.19	52.83–91.78	0	0.00
11	9	4	44.44	13.70–78.80	0	0.00
12	11	0	0.00	0.00–28.49	0	0.00
13	23	19	82.61	61.22–95.05	1	4.35
14	15	0	0.00	0.00–21.80	0	0.00
15	18	13	72.22	46.52–90.31	0	0.00
16	17	13	76.47	50.10–93.19	0	0.00
Total	280	168	60.00	54.16–65.57	4	1.43

**Table 4:** Univariable GEE analysis of potential factors associated with pestivirus seropositivity in sheep.

Variable	Category	OR	95% CI	*p*-Value
Age group	>3 years	1.00 (ref)	—	—
	1–3 years	0.46	0.27–0.79	0.004
Herd disease history	Yes	1.00 (ref)	—	—
	No	0.68	0.44–1.06	0.087
History of abortion	Yes	1.00 (ref)	—	—
	No	0.86	0.70–1.05	0.142
Parity status	Parity 0	1.00 (ref)	—	—
	Parity >1	1.44	0.89–2.33	0.139
New animals introduced to the herd	No	1.00 (ref)	—	—
	Yes	6.50	2.55–16.55	<0.001

ref: reference category. OR: Odds ratio. CI: Confidence interval.

**Table 5:** Results of the multivariable GEE model of factors associated with pestivirus seropositivity in sheep.

Variable	Category	B	SE	OR	CI 95%	*p*-Value
Age group	>3 years	—	—	1.00 (ref)	—	—
	1–3 years	−0.51	0.25	0.61	0.37–0.99	0.04
Herd disease history	Yes	—	—	1.00 (ref)	—	—
	No	−0.59	0.26	0.55	0.33–0.91	0.02
New animals introduced to the herd	No	—	—	1.00 (ref)	—	—
	Yes	0.79	0.30	2.20	1.22–3.97	<0.05

ref: reference category. B: Regression coefficient. SE: Standard error. OR: Odds ratio. CI: Confidence interval.

## Data Availability

The original contributions presented in this study are included in the article. Further inquiries can be directed to the corresponding author.
